# The Southern Dental Association, 1892

**Published:** 1892-10

**Authors:** J. M. Walker


### THE DENTAL REGISTER.

VOL. XLVI.] OCTOBER, 1892. [No. 10.

### PROCEEDINGS.

### The Southern Dental Association, 1892.

REPORTED FOR THE DENTAL REGISTER BY MRS. J. M. WALKER.

The Southern Dental Association convened in twenty-third annual session July 26, 1892, at Lookout Mountain, Tenn.

The President, Dr. Gordon White in the chair. The meeting was opened by prayer by Dr. J. Taft, of Cincinnati, Ohio.

Dr. J. Y. Crawford then, on behalf "of a few friends" presented the president with an elegant silver-mounted gavel, suitably inscribed, and held in a rich, crimson, satin-lined, plush case. The gavel, Dr. Crawford said, was made from the wood of an historic tree, grown on an historic spot, and was placed in the hands of a man of exalted character, by whom he hoped it would be cherished as a slight memento of professional and social regard.

An eloquent and witty address of welcome on the part of the Mayor, the Chamber of Commerce and the citizens of Chattanooga by Mr. H. F. Olmstead, was followed by Dr. D. R. Stubblefield, of Nashville, Tenn., who welcomed the Southern Dental Association in behalf of the State Society of Tennessee.

Dr. Geo. J. Friedrichs, of New Orleans, responded on behalf of the association.

Dr. Gordon White, the president of the association, next read his annual address, as follows:

*Gentlemen of the Southern Dental Association:*

I purpose to bring before you at this meeting several subjects of interest to our profession. The one I deem of greatest importance has been talked of, in an undertone, by many of us for a long time. We should have no secrets in our professional family—the subject is professional dignity, or rather lack of professional dignity, for the subject is too broad for me to touch on any but abuses known to all.

"Every profession has its scum," says a noted Frenchman. Alas, that those whom in the South we term good men should place themselves on a level with that scum by their methods of advertising. True, it is often only a newspaper interview that catches the eye as we glance through the paper, but it is an advertisement none the less. In the secular press of one section we find a column given the dentist who has performed, what he considers, a very remarkable operation; in that of another section a column and a half is required to properly describe the beauty and perfection of a certain piece of extensive crown and bridgework; while in still another we read not only of the wonderful inventions of our brother but also of the architecture and furnishings of his office. In one locality we find a college graduate asserting his skill in every known branch of the profession, and guaranteeing his work; in another, the familiar poem "Mary had a Little Lamb," adapted to the requirements of a dental advertisement. Such advertisements are usually accompanied by broad headlines, and not infrequently by a picture of the remarkable individual.

Gentlemen, need I tell you that members of our association engage in this reprehensible practice? Is it professional? Is it dignified? Does the profession approve it? Does it win public respect? A prominent man who for twenty years has advertised, said to me, in a recent conversation, that he did not remember a single desirable patient who came to him through his advertisements.

Why is it that we are so frequently confronted by such advertisements? Are not the schools primarily responsible for this? One reputable (?) college advertises in the newspapers and holds out as an inducement to the uninformed would-be student the fact that the dental graduate is now recognized by the medical profession as occupying the same level as the medical graduate, and further, that their graduates at once step into a lucrative practice, making in ready money so many dollars a day. Another advertises for infirmary patients and holds not even church pews too sacred for the desecration of its hand-bills. Do such advertisements on the part of the schools give the students a correct idea of the dignity of our profession?

The student while at college should live in an atmosphere of ethics. Does he? It is generally understood that there is one lecture on ethics, delivered usually by the dean at the close of the term, but perhaps not more than one-half of the students hear it.

A worthy professor calls attention to the fact that a student, as a mirror, reflects the idiosyncrasies of his preceptor. What shall we say when a graduate from a college, presumably reputable, with a certificate of the State Examining Board, locates in a town or city and at once calls attention through the medium of flaming hand bills to his "New Dental Parlors and extraordinarily low fees?" Does he not as a mirror reflect the college from which he comes? Are we not agreed that by both precept and example the colleges should sustain and increase our dignity? Are such practices (of both dentists and schools) consistent with our code of ethics? If they are, should not the code be revised? If they are not, should we not feel it our duty to report such violations? Hitherto we have been too timid to report. It is not a personal matter, gentlemen, but we owe it to our profession to aid in every way possible in the suppression of that which will drag us into the mire. The highest court of England, quite recently, held that a man who joins an honorable and registered society must strictly observe the rules of that society under penalty of forfeiture of his membership, and sustained the action of the General Council of Medical Education and Registration in removing the name of a prominent dentist from their membership because of his having advertised his business contrary to the rules of that body. The decisions of that court are a precedent for the courts of other countries. Is not the action of that council a worthy precedent for our association?

Should not the undignified practice of many reputable men of placing on their envelopes their business cards be condemned? Do they not labor under the mistaken idea that it advertises them or their business? We are professional men, not tradesmen; furthermore, I find upon investigation that in the rarest instance does a letter pass through more than four or five hands in reaching its destination and only when it fails to reach its destination is the business card referred to by the busy postman. To be sure there is nothing wrong or unprofessional in having our name and address on our envelope for the safe return of our mail, but is it not shocking to receive a letter from one of our professional brothers, the envelope of which is adorned with a cut of his wonderful inventions? Strange as it may seem, I have received such from members of this association. In a few instances I have received them with a cut of the writer on both letter and envelope, but they, I believe, were not from any of our members.

To my mind the propriety of having price-lists is questionable. There is certainly no reason why a patient may not know the cost of each operation, but the list, varying as it does from $5 to $50, practically amounts to no list. To be sure we have our rates, but you each know that in like operations the fees are rarely the same. It seems to me that it would be difficult to explain the difference satisfactorily to the patient. After all, is the list necessary? We are not in the mercantile business and do not need the advertisement. Do not our patients place themselves in our hands because they have confidence in our integrity and skill? During a practice of thirteen years I have only once been asked for a list, and during my investigation extending through a number of years I remember only one man who claimed to adhere to his list. He very frankly said that he did. If we have a list should we not adhere to it? If we do not, are we not practicing fraud and deception? Does it not look unprofessional? The "Cheap John" displays his list on his sign, the dentist of the "upper ten" on his appointment card. Is there any difference save in the fees?

In most, if not all, of our States laws have been enacted restricting in some particulars the practice of dentistry, and Boards of Dental Examiners have been appointed. These laws were enacted for what was conceived to be the protection of the public and our profession as well. They may not, and do not, fully accomplish the desired result, but they are a step towards a higher standard of requirements for the dentist, and the boards in enforcing them should have the moral support of all dentists. The boards need the support, for, while it is almost beyond belief that any one would oppose that which even tends toward our elevation, the Board of Tennessee has met with opposition.

Our fathers in 1869 organized our association for advancement in the science and dignity of our profession. Then the spirit of professional interest was stronger than the animal of self interest, and those loyal, high-minded men did not even dream that one of our memberships would ever be so debased as to be valued for personal aggrandizement. It has been said that the professions are made strong by what they include rather than what they exclude. Let us then include so much love for our grand profession, such high, pure aims in its practice, so much enthusiasm for its advancement, that there will be no room for any unprofessional act or thought. Let us work to an ideal, and let that ideal be as high as finite conception can reach.

Much has already been said in regard to a home for the Southern Dental Association and a permanent committee on a "Dental Chautauqua" has been appointed. The idea, as I understand it, is to erect at some desirable summer resort a building in every way adapted to the needs of the association, and where year after year the meetings may be held. There is much that is desirable in this plan, but is it practicable? It means the outlay of a large sum of money without any return, a dead weight for the association to carry. Furthermore, men will not go to the same place year after year.

If the association will have a home, let it, by all means, be located in some central city and so constructed that a part can be rented, yielding sufficient revenue to pay all expenses. So located "The Home" will be an object of interest to all dentists passing through that city. Besides, there will always be a number of resident dentists to keep up the interest.

My preference is not a Southern but a National Home, and, as has been suggested, a national museum, located in Washington or some central city, where from all parts of the country we can send our treasures. Why not unite with the other societies and build a home that will be a credit to our profession and establish a museum that will fittingly preserve our history for this and future ages—in other words, a monument to the dental profession of America.

In 1890, at our meeting of the association in Atlanta, it was suggested that we be represented at the World's Fair in 1893. The American Association took up the suggestion and a committee of fifteen has been appointed by the two societies, which committee will meet during the present session. The work of organization is far advanced and the World's Columbian Dental Congress will be held August 17 to 27, 1893, in Chicago, Ill. Let us not forget that it was our suggestion and that as such it behooves us to give the committee all the support they expect from us. Certainly they have a right to expect our presence, and so far as possible we should attend this congress.

At our last meeting there was a resolution to the effect that the constitution be so changed as to provide for the election of officers at a much earlier hour, so that the newly-elected president might have the opportunity to make his appointments. This idea is excellent, and I would suggest that a section on Orthodontia be created. Properly it does not come under the head of any existing section, but is separate and distinct.

It would be well also to thoroughly revise the constitution. There are some defects not necessary to allude to here, which a committee would readily detect, and of which all the ex-presidents are aware. The principal one is its vagueness in setting forth the duties of the officers and committees. I do not make this last suggestion to bring about the discussion of the constitution, for we wish to embody nothing new, but simply to make plain that which we already have.

I have called attention to these things, gentlemen, because of my deep interest in the continued advancement of our profession and the preservation of its dignity.

In the name of those who have shed luster on that profession let us be faithful to our sacred trust, transmitting to those who will succeed us an honorable record of duty faithfully performed.

On motion of Dr. J. Y. Crawford, a committee of three was appointed to consider the President's Address and present it for discussion. Drs. Chisholm of Alabama, Lawrance of Georgia, and Marshall of Arkansas, were appointed on this committee.

On motion of Dr. M. C. Marshall, Dr. Augspath, one of the founders of the association, but who has been absent from its meetings for many years, was reinstated into full membership on payment of one year's dues.

Dr. W. W. Walker, Chairman of the Executive Committee of the World's Columbian Dental Congress, made a report of the work accomplished. He said that it would be impossible in the brief time available to give any detailed account of what has been done, is being done, and is intended to be done by the various sub-committees which have been organized and are hard at work.

As an illustration he would only say that Dr. Taft, Chairman of the Committee on State and Local Societies, is now in touch with every reputable dentist in America. This alone indicates the vast amount of work accomplished by one committee only. The Committee on Finance, of which Dr. L. D. Shepard, of Boston, is the Chairman. Without wealth at command the work of the organization would amount to nothing. Money must be had to run it. He hoped that all would become active members, and go down into their pockets as deeply as their purses would allow. He hoped that nothing would ever occur to mar the friendly feelings now existing between the different associations, but that all would meet in Chicago in 1893, joining hands as a happy band of brothers, working together for the general elevation of the profession at large.

At the afternoon session the Committee on the President's Address reported their indorsement of the views presented on the subjects of Dental Ethics and of the functions of State Boards, but begged respectfully to differ with him on the matter of the Dental Chautauqua movement, admitting, however, that the question as to the feasibility of locating at all was an open question but worthy of mature deliberation. The subject of revising the constitution they deemed of the utmost importance, especially in the matter of more clearly defining the duties of committees, the present form being vague and indefinite.

The President's Address was then discussed at some length by Drs. Catching, Chisholm, R. R. Freeman, McKellops, J. Y. Crawford and Peabody.

Dr. Catching thought the colleges could not be fairly held responsible for the actions of their graduates. Dr. Freeman held the contrary view. He said that man was naturally an imitative animal, and from childhood up endeavored to follow the example of those to whom they look. The application is evident. Dr. McKellops spoke of the lamentable lack of business qualifications in the average dentist. Every man, even if he be a dentist, must make money for the support of his family. For this reason he should associate more with business men. He should take an interest in public affairs, and be a good citizen, thus elevating himself both from a business and from a social standpoint. He did not favor the idea of a permanent home for the association, which would soon become an old idea, and men would stay at home, benefitting neither themselves, the profession nor humanity.

Dr. Crawford spoke very earnestly upon the violations of the code of ethics, especially in the endorsement of patent nostrums—not alone by members of the dental profession, but especially by the clergy, who, while denouncing drunkenness, sign their names, and even permit their portraits to be appended to endorsements of patent medicines known to consist largely of alcohol or whisky. He believes that the injuries from patent nostrums are more far-reaching and baleful than can possibly be ascribed to the rum traffic, notwithstanding the $900,000,000 annually expended on ardent spirits. As a practical fact no man ever received benefit in a professional way through improper advertising; sooner or later it redounds to his injury. He hoped to see the day when the ethical code would be so pronounced and so strong that neither the professional man, preacher nor editor would dare to be a party to a fraud simply because he was paid for it. He said that he hoped the subject of a Committee on Orthodontia, recommended by the President, would be brought up and acted upon. There was not one other question in the whole subject of pediatries of so much importance to the human family as Orthodontia, and he hoped an active committee would be formed.

Prof. Peabody said that no legislature could legislate a man into a gentleman. Men who boldly blow their brazen bugle-blasts make us ashamed of our profession. But we cannot overcome this hydra-headed monster though we may decry it most heartily. We feel ashamed to acknowledge our profession because of its prostitution. We must educate the public to understand that from such men they will not receive value for services rendered. If they put themselves at the mercy of charlatans they can only expect future misery—they will get only what they pay for.

Dr. B. Holly Smith, of Baltimore, read an elaborate report, as Chairman of the Committee on Dental Education, of which the following synopsis is given. While recognizing the debt of gratitude due to the dental laws and efficient dental boards of the different States, constituting a complete and adequate machinery adapted to the work of excluding imperfectly prepared candidates from the profession and forcing constant elevation of the standard of requirements for entry into the profession, he said that individual and collateral questions were arising whose adjustment involves the rearrangement of some of the machinery that has been provided for the protection of the profession and the public. For decades and centuries, the history of progress itself has been the history of some famous colleges—the story of its development, its conservation of what was valuable in knowledge, its propagation of what was alive and fructifying in thought. The similar influences of our dental colleges should not be underestimated. The position of Examining Boards is the direct outgrowth of a law designed not primarily for the elevation of the standard of dental education—that being an indirect and incidental effect—but for the protection of citizens from the evils and injuries which they may receive at the hands of incompetent and ignorant practitioners. All dentists are deeply interested in the proper execution of such laws, for as citizens they desire the amplest protection, and as professional men they desire to be spared the shame and mortification attendant upon the blunders and mishaps of the ignorant and incompetent. While it is a source of pride and congratulation that the execution of these laws has been entrusted to most competent and efficient Boards of Examiners, who have thoroughly and conscientiously discharged their duty—that the gentlemen composing these boards have recognized this double duty to the State and their profession, and have discharged their functions in a manner calculated to protect the interests of the one and to advance and elevate the standard of the other—yet there is nevertheless some slight inharmony among the elements that should work together for the good of all concerned, viz., the citizens (or patients), the profession, the colleges, and the student. As citizen to citizen we have discharged our responsibility when we have afforded protection from quacks and charlatans in granting license only to those possessed of the requisite knowledge and skill. In this matter the power of boards is entirely discretionary; its exercise may become either perfunctory or prohibitory. The standard must necessarily be shifting, while examinations alone can not always establish the fitness for or unfitness of the candidate. He may be unable to discuss theoretically what he understands practically, or to *say* well what he can often *do* excellently. If a test such as an Examining Board can give is to be accepted as efficient and satisfactory, certainly a test extending over three years and covering every variety of instruction and practice he is likely to meet with in practice, must be more so. The conclusion then is that "a diploma from a competent faculty, granted after three years of faithful study and initiatory or student practice, should have a preponderating weight in the establishment *vel non* of a candidate's fitness to practice his profession." How can we expect the diplomas of American Colleges of Dental Surgery to be honored in Europe when they are not properly estimated in our own country? As professional men, the elevation of our profession is the aim towards which all our efforts are bent. We have had a hard fight to secure proper recognition among the learned professions, as seen in our recent classification among tradesmen in the collection of census statistics. This incident shows how continually we must be on the alert, and that we have those in our ranks who are both watchful and progressive. The profession rightfully claims from the colleges an adequate and thorough course of instruction, competent and scholarly instructors, and honorable, conscientious labor. And the pledge of the colleges—a sacred and solemn pledge—is to eliminate, by thorough and conscientious instruction, the immature and unadapted from the ranks of those seeking entry into dentistry. The responsibility of colleges must of necessity be higher and greater than that of Examining Boards. The former undertakes to prepare, the latter simply to exclude. They incidentally guard the profession while protecting the public at large. Their work for the advancement of the profession, however, has been none the less distinguished because indirect and incidental. That the student has just claims upon us no one will deny, and while we gently require of him ability, adaptability and adequate preparation, it is just as much a matter of convenience and of duty to make sure that we do not, whether through pedantic blundering or honest errors of judgment, exclude the capable, the earnest, the ardent, the enthusiastic, the prepared, for these are they whose work shall reflect honor and glory upon our loved profession.

Many a young man, purposing to enter dentistry, is now, in his present relation to the unrelated and unconnected college and Examining Board "between the devil and the deep sea." While convinced that the proper place to study dentistry is at some reputable dental college, yet right before his eyes he has college graduates whose diplomas, in the hands of an all-powerful board, have been as "clay in the hands of the potter," neither a defense nor an honor. Why should he not say: "What is the use of wasting my time and my money in going to college and attempting to prepare myself by a three-years' course of study and practice for my life-work, when the evidence of that work weighs as nothing with the examiners?" And the disgrace of failing! The poor plucked wretch may conclude, with some justice: "I don't see how that examination is a fair test of my ability to practice dentistry. There were many important and essential questions I did not know and was not asked about. This examination may have shown this board what I don't know, but *may I be elevated* if it showed what I do know?"

All this must conduce to undervaluation of systematic training and continued study, and opens the door to irregularities in proportion, and a consequent lowering of grade in requirements, skill and science. As an effort towards the adjustment of these somewhat incongruous elements with a helpful and healthful harmony, the final proposition made by Dr. Smith is briefly, the examination of candidates for graduation by the Examining Board of the State in which the college is located, as severe as the board may choose to make it (the college being competent and willing to meet the test), the student feeling when he has graduated under these test conditions that he has earned the right to go forward in his profession, earning, in the daily exigencies of his work, more than either colleges or boards can teach him. Freed from the thought of examination after examination hanging over his head, he will rely upon faithful study and adequate preparation rather than an uncertain kind of ability to perform those mental gymnastics miscalled examinations.

This subject was discussed by Drs. Chisholm, Browne, of Atlanta; Wright, of South Carolina; Turner, of North Carolina; Jones, of Florida; Young, of Alabama, and others, being continued at the night session of the first day.

Dr. E. S. Chisholm spoke of the necessity which had compelled the enactment of dental laws and the creation of Examining Boards, because of the inability and poor preparation of many of the earlier college graduates, and in the vast improvement in late years, which justifies the hopes that it will soon be possible to admit all three-course graduates to practice without examination.

Dr. W. C. Browne said there was no clash between the boards and the colleges, the influence of the former being wholly towards a higher education, uplifting the profession and stimulating the colleges to greater endeavors. When the boards report students deficient in certain lines the colleges work up on those lines and students come out better qualified.

Dr. V. E. Turner thought it impracticable for students to be examined before the Board of the State in which such college is situated, because he does not always propose to locate in that State, and the laws of the different States are not the same. A student after he is thoroughly prepared and has stood his college examination should have little difficulty before a State Board. When fresh from college he will probably be "up" on all technicalities.

Dr. J. N. Jones thought the laws should be uniform in all the States, and the license of one State Board should be good in all the States; that there is too much red tape about the business. A man may cram and pass an examination without being qualified to practice dentistry.

Dr. R. C. Young considered the three-term course of the colleges the most gigantic step taken by the profession, and that it was brought about entirely through the influence and efforts of the State Boards. He spoke of the extreme severity of the Alabama State Board, and thinks that in the course of a few years young men will come from the college so thoroughly prepared that Examining Boards will be such only in name.

On motion of Dr. S. B. Cook the President's Address was ordered published in a daily paper of Chattanooga for the instruction and enlightenment of the people, who ought to know something of what we are doing.

In closing the discussion at the night session Dr. B. Holly Smith said that his friends desired that he should put himself right, as a false impression had been created, namely, that as a college man he had expressed opposition to the Examining Boards. This was a false impression as he had intended nothing disrespectful or unkind. They had done much that was laudable and commendable, and had endeavored to harmonize and establish proper relations between the profession and the colleges. He would rather have those who had been under his instruction pass before the most strict and rigid board than have them go out unprepared. He said: "When I have worked with a man for three years I know what he knows, and though he may become paralyzed through nervousness and fail before his State Board, that does not prove that he is not fit to practice dentistry.

Dr. E. S. Chisholm said the men on the State Boards did not claim to be teachers, and perhaps they do not always know how to formulate questions, but they honestly try to bring out the knowledge of the applicants to the best of their ability. If a young man cannot tell whether or not a child has deciduous bicuspids, or thinks it will take about five years for a broken off corner of a tooth to build itself up again—such a young man is not properly prepared even if he is a college graduate. Stricter requirements we need on chemistry and etiology, and the attention of the colleges should be directed to these two branches especially.

The report of the Committee on Prosthetic Dentistry being called for it was found that the chairman was not in attendance and had furnished no report, neither were there any papers before that section.

Dr. R. R. Freeman, Nashville, Tenn., said what he had designed to form a portion of the chairman's report was intended to be woven in as a part of the whole. He asked the question, When, from the ravages of decay, the whole, or a portion, of the organs of mastication have been lost, what shall be done to restore the loss, and bring about the nearest possible approach to natural function? The most important step is to suspend, as near as may be, the devastations of the destroying agents by removing them, or by counteracting their influence, affording nature a chance to assert her supremacy. After restoring the parts to a physiological condition, the mechanical arts aid us to furnish a substitute that will harmonize in appearance, feeling and function with the surrounding organism. In the construction of these substitutes there are mechanical laws which must be understood and applied. While the principles of mechanics are unchangeable we must also remember that we are dealing with living structures subject to physiological and pathological changes.

A clear plan must first be outlined by intelligent study of the case of what it is desired to accomplish. In the accomplishment of your plan there are a few minor points to which your attention may be profitably directed. In the selection of an impression cup never hesitate to cut, bend, or twist one, or even construct one especially for the case in order to make it conform to what you desire. Never let the impression material extend beyond the margins of your cups, or you will have imperfect margins. The ideal cup should come within the thirty-second part of an inch of touching every part on which the body of the plate is to rest, the palatal border resting in easy contact. When the cup, with the impression material, has been placed in position in the mouth, the cheek should be drawn out, and the lips first down and forward and then pressed upward, bringing the plaster firmly against the alveolar border, before the plaster begins to set. While the cup is held up with sufficient firmness to prevent displacement there should be an apparent movement as though to draw it downward and outward so that the muscles will be relaxed. If driven up too tightly the tissues will be hard and compressed unnaturally. Of course it cannot actually be held up and drawn out at the same time, but the pressure should be exerted as though it were about to be drawn out.

When the teeth are set the plane of mastication should fall lower in the rear than in natural occlusion, and there will be less likelihood of displacement during mastication, and the tendency of the plate will be to become firmer with use. A cup for the lower plate should have deep wings extending below the line where the plate is to rest to avoid enfolding the mucous membrane, and pressing away the tongue at its base. A lower plate should always extend well back and up along the ramus to prevent wabbling in the mouth. A well defined ridge extending around the border, say within one-eighth of an inch of the gum margin, will assist greatly in retaining it in position. This is easily made, after the teeth are waxed up, by using a piece of wrapping twine which has been saturated in wax, made to adhere just where you want it by a puff of the blowpipe flame. When reproduced on the plate it will afford a line on which the lip will take hold, causing it in many cases to adhere with great tenacity to the jaw.

The president said he hoped that in the absence of papers the subject would be discussed, as it had been greatly neglected in the association.

Dr. A. P. Johnson, (S. C.) exhibited the model of a device for keeping the lower plate in place.

Dr. Browne (Atlanta) instead of pressing and holding up an impression cup, tells the patient to bite it up and hold it steadily in position himself, thus relieving both patient and operator from the usual uncomfortable position while waiting for the plaster to set. When the tissues are very hard he lines a portion of the surface—varying from the dimensions of one air chamber to the whole surface in extreme cases—with soft, flexible English rubber. In finishing it up it is scraped until the color of the flexible rubber can be seen through the hard rubber surface which is necessary for the polish. This method will make the plate adhere in the most difficult cases. It is equally valuable in lower plates.

A number of satisfactory cases of the Marshall Anchor plate were cited by members of the association who had been using it since the patent was given to the association by Dr. Marshall a year ago. This method was discussed by Drs. Morgan Adams, Catching, Thompson, of Atlanta, Dotterer, A. P. Johnson, Chisholm, Marshall and others, the plate having given universal satisfaction, especially when anchored to one tooth only.

Dr. Geo. Evans (New York City) described his method of making a double crown or cap. After adjusting a gold crown to the tooth in the mouth, he removes it, and winds around it a piece of paper which he fastens with a wire or string. Into this he pours Melotte's fusible metal, making a die on which to make his telescoping cap. On this he winds No. 30 to 32 pure gold wire, binding one end over the top of the crown. This he solders; soldering on the outside a narrow strip of No. 32. Any danger of solder flowing inside of the telescopic cap can be obviated by painting it with whiting. When all is finished, a little fusible metal is melted in a cup and the crowned die dropped into it, when the gold crown will float and can be picked out. If any of the fusible metal still remains, the crown can be immersed in nitric acid, which will remove the last particle. He finds this a clean and accurate method.

Dr. Beach (Clarksville, Tenn.) adapts a band to the curve of the crown of a tooth to which a plate is to be attached. On this band he telescopes the band of the plate. Removing the band from the tooth he fills it with plaster and wraps around it No. 40 tin foil, marking where the telescoping band is to come. With the tin foil he thus makes a pattern by which to cut the gold. When the gold is soldered and burnished down on the crown it makes a very perfect fit, and is done as quickly as the telling. A lug or attachment for securing it to the plate is soldered on afterwards.

Dr. Thompson (Atlanta) after cementing the crown on the tooth gets a metal model of the tooth with the crown on. To this he adjusts a 22 carat band which he slips off and solders, bending the ends back in opposite directions giving good attachment to the rubber of the plate. The bands fit the teeth perfectly, and yet slip off readily. He casts a tin model directly from the impression, and if any accident happens to the plate there is no necessity for taking another impression. He uses pure block tin which melts easily and does not tarnish.

Dr. S. B. Cook (Chattanooga): To make a perfectly smooth metal model, smoke the plaster impression in the flame of a tallow candle and the metal will flow perfectly smooth.

Dr. Browne (Atlanta) uses tinner's solder instead of pure tin, as it is more easily obtained. He takes his impression and sets it on the stove at the same time with the ladle of metal. By the time the metal is melted the plaster will be dry enough. The metal must be poured in slowly as it hardens dropping it in and building it up the sides of the flask.

Dr. Cook in case of undercuts or overhanging margins lays strips of isinglass (mica) across with the ends sticking out. This divides the model into sections which can readily be put together in position.

Dr. Freeman to support a telescoping band when you have sound roots a Richmond or Logan porcelain crown may be used, attaching the plate by clasps to the porcelain crown.

The morning of the second day was set apart for clinics. The election of officers was held at the afternoon session with the following result: President, Dr. B. Holly Smith, Baltimore, Md.; First Vice-President, Dr. R. K. Luckie, Holly Springs, Miss.; Second Vice-President, Dr. S. B. Cook, Chattanooga, Tenn.; Third Vice President, Dr. L. P. Dotterer, Charleston, S. C.; Corresponding Secretary, Dr. D. R. Stubblefield, Nashville, Tenn.; Recording Secretary, Dr. S. W. Foster, Decatur, Ala.; Treasurer (re-elected), Dr. H. E. Beach, Clarksville, Tenn.

Dr. J. N. Crouse (Chicago), President of the Dental Protective Association, was then introduced by the President and proceeded to address that body in the interest of the association he represents, this being his first visit to the Southern Dental Association, and his first trip so far south in this country.

His address was listened to with close attention and at its close a vote of thanks was tended to Dr. Crouse for the work he has done for the dental profession, and the hearty and earnest support of the Southern Dental Association pledged.

The subject was discussed by Drs. Dotterer, McKellops, Freeman, Peabody and others, and at its close, on motion of Dr. W. H. Richards (Knoxville, Tenn.), Dr. Crouse was elected an honorary member of the association with a rising vote and applause.

On motion of Dr. R. K. Luckie a committee of three from each State was ordered appointed to bring the Dental Protective Association to the notice of members of the profession throughout the South with the expectation of doubling, if not trebling the membership when the territory is properly canvassed.

Thursday morning was devoted to clinics. At the afternoon session Dr. B. Holly Smith addressed the association on the subject of the Wilcox Bill—a bill, he said, constructed to compel dentists to give in statistics as ordinary mechanics and manufacturers, whereas they are to be classed as professional men of scientific attainments. He related the history of the fight that has been made against the bill, and the result obtained—namely, the agreement on the part of the Chief of the Census Bureau that no dentist, surgeon or lawyer shall hereafter be called upon for such statistics.

Feeling a jealous regard for the rights of the Southern Dental Association, he felt that she should have the honor of joining the American Dental Association in meeting the expenses of this fight for rights by which we are equally benefited.

The following protest and resolutions were read and adopted:

A PROTEST AGAINST THE CENSUS CLASSIFICATION OF DENTISTS AS MANUFACTURERS.

WHEREAS, It has come to the knowledge of the members of the Southern Dental Association that House Bill 7696—known as the Wilcox Bill—passed the House of Representatives June 4th, 1892, and is now before the Senate for action, and that said bill provides for the collection of statistics from incorporated and unincorporated companies, firms, associations or *persons engaged in any productive industry*—and according to the classification of the superintendent of census of 1890, appointed by the Secretary of the Interior, dentists are classed as manufacturers.

*Therefore*, Be it resolved that we protest against the collection of statistics from dentists as manufacturers, or as the bill has it, "persons engaged in productive industries." Dentists are not manufacturers in any sense, not being engaged in any form of fabrication, or the sale of any product having a merchandisable value. The laws regarding the practice of dentistry in the various States and Territories and in the District of Columbia, distinctly recognize dentists as professional men, and the attempt to collect statistics from dentists as manufacturers will not only be an injustice to dentists and their patients, but useless to the government as showing the productions of a class of men not engaged in manufacture, and an erroneous interpretation of the spirit of the bill.

On motion of Dr. B. Holly Smith a committee of three was appointed to correspond with the chairman of the committee which has accomplished this work, and with the committee of the American Dental Association to raise funds for defraying the expenses of the fight.

Dr. W. J. Barton (Paris, Texas), Chairman of the Committee on Pathology and Therapeutics read the report of that committee.

ABSTRACT OF DR. BARTON'S REPORT AS CHAIRMAN OF THE COMMITTEE ON PATHOLOGY AND THERAPEUTICS.

Dr. Barton reported, from this section, a paper on the Care of Children's Teeth, from Dr. Foster, Decatur, Ala.; a paper on Nitrous Oxide in Cardiac Weakness, by Dr. Sharpe, Knoxville, Tenn.; a discussion on the Therapeutic Value of Electric Currents, by Dr. J. S. Marshall, Chicago, Ill., and a discussion on the Presistent Effects of Medicaments Sealed over the Dental Pulp, by Dr. B. H. Catching, Atlanta, Ga.

Dr. Barton, in his report, spoke of the advisability of cultivating the fraternal relations which ought naturally to exist between dentists and physicians. To ignore and disparage the physician is to destroy the growing recognition of dentistry on the part of the general practitioner, for while it is true that dentistry is technically recognized as "a specialty of medicine," it yet remains to be made practically so.

Dr. Barton mentioned as among the exciting causes of pathological conditions of the gingival borders and the peridental membrane, the ill-fitting and impinging bands in crown and bridgework, especially where more regard is paid to appearance than to healthful conditions. Another cause of pathological conditions is found in too great pressure from rubber dam clamps, producing a diseased condition about the necks of the teeth, which soon develops into caries.

Pathological conditions of the peridental membrane may result from the animal matter remaining in the tubuli of the dentine after pulp removal, indicating the necessity for extra precautionary measures in the way of antiseptic treatment and permanent antiseptic dressings. The pathological condition known as Sensitive Dentine may be an inflammatory condition resulting from the loss of enamel or other protecting tissues, or from inflammation of the pulp. A metallic filling may not be, and probably is not, in itself a proper protection in these conditions. Permanent oily dressings and varnishes, and other non-conductors, are indicated, and the use of nitrate of silver where fillings are not applicable, or where the teeth are too sensitive for proper excavation, especially in the teeth of children.

Dr. S. W. Foster (Decatur, Ala.) here read a paper on Children's Teeth. He dwelt upon the pathological conditions of dentition and the treatment of the deciduous teeth. It is to the proper care of the deciduous teeth that the permanent teeth are made to subserve the purposes for which they were created and the arches filled with properly occluding and perfect permanent dentures. This accomplished the dentist may feel that he has achieved a glorious success, and it is the exception when such results cannot be reached with proper treatment. And yet it is alarming to note the indifference with which the majority of our dentists have treated this subject, though there seems now to be a progressive movement along this line. He said, if by adding my mite I can induce some who have treated this subject with indifference to become more earnest in the care of the deciduous teeth I shall feel that my services have been well rewarded. It is gross neglect on the part of the dentist to fail to disseminate among parents a knowledge of the pathological changes accompanying dentition, and the therapeutical and surgical treatment necessary, and yet, who among us fails to have presented daily children whose mouths are filled with decayed teeth, chronic abscesses, suppurating sinuses and vitiated secretions—seething pits of filth, generating bacteria and disease enough to break down the most robust constitutions. What are we to do to bring about a change in their conditions? First, we must have the perfect co-operation of the parents. Children should hear no more tales of the cruel tortures inflicted by that horrid dentist, and they would no longer come to us as into the hands of the executioner. We must give these little patients time and consideration, deal with them by persuasion, gentleness, and yet firmness; make them feel that we are their friends, and that what we would do for them is for their good, and the victory is soon won. Though we cannot control pre-natal conditions we must educate the people, making parents realize their duty to their children from a pathological and therapeutical standpoint. Insist upon it that our public schools teach the rising generation the pathological as well as the physiological changes to which the teeth are subjected. Could such a course of study be given the children of to-day the parents of the future would feel the necessity of caring for the children's teeth. The greatest number of deaths occur among children during the period covered by first dentition, growing more out of the condition of the oral cavity than from any other cause, for though our most noted authors hold that dentition is strictly a physiological process, yet it is an exception when we do not find pathological attendants. Such conditions during dentition as flushed cheeks, restlessness, loss of appetite, fever, swollen and inflamed gums, otitis, vomiting, diarrhœa, convulsions, often culminating in death, cannot always be a mere coincidence, but must to a great extent be recognized as the pathological attendants of first dentition. Pure, healthy food is the first essential prophylactic measure. Unless imperatively prohibited by circumstances the child should be fed entirely from the mother's breast until dentition is completed, that is, until we have an unbroken row of teeth, above and below. It should have an abundance of free exercise, fresh air and sunshine, and by all means, the oral cavity as well as the entire body kept scrupulously clean. With these simple precautions we may look for well-organized teeth, freedom from constitutional disease and that very prevalent trouble the, urinary diathesis. He said, when the gums are swollen and inflamed I am an ardent advocate of the gum-lancet, from my own observation of the good results following its use. Perfect cleanliness is the great preventive of decay. Could the mouths of children be kept in a sterilized condition until the age of fourteen years, and the idea of oral cleanliness instilled into their minds, caries in after years would be the exception, not the rule. In placing the mouths of children in a sanitary condition all stains should be removed by the use of a sott brush and pumice, and I have seen good results follow the application of pure wood creosote, after polishing, as recommended by D. Nael—the beechwood creosote—not the commercial article. Never remove deciduous teeth prematurely. Treat and fill with some of the plastic filling material, and retain them till the natural time for shedding through the absorption of the roots.

If we were more fully impressed with the fact that the deciduous teeth were designed to fulfil the same mission for the child as the permanent set for the adult, we would be more considerate in their care and preservation. There is no more important element entering into the development of the growing child than a perfect digestive apparatus, and this it cannot have unless mastication is good, and no child can properly masticate without all its teeth, or with decayed and aching teeth. The temporary teeth cannot be extracted previous to the development of the sixth-year molars without a resultant disarranged and crowded arch when the permanent teeth appear. In the language of another: "It matters not what an unappreciative world may say of us, our work will certainly bear leaf, flower and fruit, which bursting forth, in the end will show in part how far reaching is the range of usefulness to which the dentist may justly lay claim."

Dr. S. P. Sharpe (Knoxville, Tenn.), read a paper entitled "A Plea for Nitrous Oxide in Cardiac Weakness." Admitting the necessity of protection from shock incident to sudden pain or even excitement in the case of patients suffering from any form of disease of the heart, Dr. Sharpe claims, after an experience of thirty years in its use, that nitrous oxide furnishes such protection and is therefore an agent that should be hailed with delight by suffering humanity. He said out of the vast number who annually take nitrous oxide there are not a few who are not more or less afflicted with disease of the heart, yet death is almost unknown. This being the case, ought it not to be proof to any unprejudiced mind that the statements of its enemies are without proper foundation? At a low estimate, I have administered it to more than a hundred coming from physicians who had pronounced their hearts too much affected for any anæsthetic, yet I have never seen the first distressing case from its use in a practice that consumed more than forty thousand gallons of it each year.

But granting that a possible death might occur from its use I claim that the danger is so remote that in my judgment we would be likely to have almost as many deaths from shock in proportion to the number of operations, if we used nothing at all. He concludes, therefore, that the danger, if any, is not commensurate with the benefits derived from its use.

Dr. John C. Storey (Dallas, Texas), read an address on the subject of the Dental Chautauqua for the S. D. A. Dr. Storey would not, as so many propose, establish the home of the association in some far-away mountain gorge, or other health resort, but rather in one of our great cities. He would have it a chartered institution, with buildings sufficiently spacious for lecture halls, operating rooms, laboratory, museum, etc., with an auditorium sufficiently capacious for commencement occasions, association meetings, etc., with a faculty competent to teach medicine in all its branches; sufficiently endowed to free its members from all labor save that of teaching, giving their whole time to the school, with no practice save in consultations—a diploma from this S. D. A. school to constitute life-membership in the association. This school should be open at all times to members of the association who might wish to "rub off rusty spots," or acquire the newest ideas—constituting in fact a true post-graduate school. The annual meetings of the association he would have held during the last month of the regular session of the school.

Further details of the plan were outlined at length by Dr. Storey looking towards the establishment of "an institution of learning such as no student has ever been privileged to enter, and to be an alumnus of which would be an honor to which no graduate has ever yet attained."

Dr. John S. Marshall (Chicago), addressed the association, from notes, on the subject of Electricity as a Therapeutic Agent. He said that while electricity had a wide range in medicine and general surgery, in dental surgery it was lessened probably because its application was not so well understood. In his own experience he had found it had a specific value in the treatment of hypertrophied conditions of the pulp and dental membranes, of benign tumors of the gum and the alveolar process, using the continuous galvanic current, or the Farradic current as indicated by the conditions. The positive current produces anæmia and depletion; the negative, congestion and plethora; the Farradic current stimulates the absorbent organs, reducing hypertrophy and neoplasms. He uses the appliance of Dr. McIntosh, of Chicago. A little spring of bent wire with a small cup at each end fastened on the inside, and a copper wire running through. A small bit of sponge or absorbent cotton is placed in each of the cups, which are placed on either side of the gum or tooth, the electrode from the positive pole of the machine being attached to the opposite wire. The negative electrode is held in the left hand, or applied at the base of the brain. A galvanometer is used to measure the strength of the current, not more than three-fourths of a milliampere being used at first.

In hyperæmia of the pulp this is very successful, continuing the application from ten minutes to half an hour and very gradually increasing the current. The current should be light, but stimulant, using the continuous galvanic current. The Farradic current being an interrupted current would cause shock and increases hyperæmia, and is to be avoided. Such remedies as aconite, iodine, etc., may be used through the sponges, their action being hastened through the current, and they are more readily absorbed and rendered effective than by mere topical application. The Farradic current is used to test teeth for vitality. It is often extremely difficult to say positively whether a tooth is alive or devitalized. There may be no sensation through the cusps; the tooth may respond but vaguely to cold water or hot water, but if the Farradic current is applied through the positive pole you can make no mistake. A live tooth cannot stand one-half a milliampere, while on a devitalized tooth the strength of the current can be increased to two or three milliamperes. He said, I cannot now go into the treatment of tumors and other uses of the electrodes, but you will find it has a very useful place in your armamentarium.

The subject of Pathology and Therapeutics was then declared open to discussion.

Dr. Barton desired to call more especial attention to the use of nitrate of silver in the treatment of children's teeth, and in arresting caries in the teeth of adults. He said all that has been claimed for it has been fully sustained in his experience. His experience was confirmed by that of Dr. Beach, Dr. Jones, of Fla., and others. Drs. Nael and Chisholm claimed that this was simply an old idea revived. Dr. Richards said that the old practice was to use it for reducing sensitiveness but not with the object of arresting caries.

Dr. Jones said that he had used it with the former object, but with the latter result to his surprise and gratification.

Dr. Catching (Atlanta) spoke of the effects of persistent medication on the dental pulp. When the nerve is not quite exposed a paper saturated with carbolic acid is often laid over the thin layer of dentine next the pulp, and the tooth immediately filled. This is bad practice, for the result is just what we want to avoid —the death of the pulp or a weary struggle for existence. Other means may be used to disinfect, but do not swab in carbolic acid, and the remedies used should not be confined in over a nearly exposed pulp.

Dr.———— said the action of the nitrate of silver in arresting decay was through the affinity of the nitric acid for the water in the tooth, the silver being liberated and filling the tubuli of the dentine. Subject passed.

At this point a paper was received from Dr. Ottofy (Chicago) too late for consideration by the section of Dental Education.

On motion it was now read. The paper was entitled "Post Graduate Study" of which an outline is given here. Admitting the fact that the dentist who is not absolutely progressing is certainly retrograding the necessity is obvious of some means of improving the head and the mind of the dentist in active practice while not removing him from the scene of his labors or interfering with his daily practice necessary for the support of his family. The Chautauqua system of home reading offers a solution of the difficulty, and similar courses of reading have been organized for the higher education of the dentist.

Arrangements have been made with the publishers for procuring books at lower than market rates, and membership in the association entitles the reader to these rates and also syllabus outlines of the contents of the book with instructions to be carried out each month. Any desired information or elucidation of obscure points will be furnished by the authors of the books selected, or by the officers of the association. At the completion of each course written examinations are required and certificates granted. The courses A, B, C and D, will include text-books graduated from the raw recruit, fresh from the dental college, to the oldest practitioners, and will include anatomy, physics, chemistry, bacteriology, dental jurisprudence and the highest of dental literature. The examinations will probably exceed anything heretofore known, and men whose reputations are established as scientists may tremble before Yale, Harvard, or even Oxford, Cambridge and Heidelburg examiners. Through this movement the elevation of the intellectual status of the profession must be attained and the effects will be felt in generations to come.

In the absence of the chairman of the committee on Operative Dentistry (Dr. Rollo Knapp) Dr. Nael read a paper sent by Dr. J. H. Allen, entitled "Some of the Causes of Failure in Gold Fillings," of which a brief synopsis follows:

Dr. Allen emphasized the importance of properly shaping a cavity and in certain cases of getting a tooth into its proper position before filling, illustrating the latter idea by describing a case in which a second superior bicuspid having a compound proximal cavity on the anterior and coronal surface extending well toward the gingival margin, and having been in this condition long enough for the first bicuspid to fall back until the contour was lying somewhat in the cavity, the interdental space being nearly obliterated and the gum hypertrophied by compression. This case was presented at a clinic. The operator having removed the hypertrophied gum tissue, cut away a large portion of the tooth substance and adjusted the rubber-dam, asked the question of the bystanders, "How long would it take you to fill that cavity?" Various replies were made, ranging from one hour up, for the insertion of the gold in the already prepared cavity. Dr. Allen replied "three weeks"; saying, "That tooth is in no condition to fill until it occupies its normal and correct position in the arch, relieving the pressure on the interdental gum and alveolus, which will be augmented as it now stands by the separation for space and the subsequent finishing of the filling. This crowding of the parts will cause the death, not only of the intervening gum tissue but the destruction of the interdental alveolus by absorption, and you will have a worse condition, resembling pyorrhœa alveolaris, besides which the filling will probably fail at the cervical portion, and you will have a worse condition than before." In such a case Dr. Allen advocates separating with rubber, following it up by pledgets of cotton until the tooth was restored to its normal position, and holding it in place by means of phosphate between the teeth, so placed as not to impinge upon the gums in the least, filling only after all soreness was gone. He would fill with gold, using soft foil for two-thirds of the cavity, finishing with hard foil which can be finished and polished with plug finishers and then polishing tape, without cutting away any of the contour of the cohesive part of the filling. He would not use a matrix preferring rather to build against the other tooth if necessary and cut it away subsequently.

In cases when the cavity extends so deep towards the cervix of the tooth as to very nearly go beyond the enamel, the enamel will be found to be so very thin that the least downward pressure will cause minute particles to be broken away leaving little rough places. In such cases he would carry the cavity deeper and prevent failure at this point.

Excessive malleting is the next cause of failure mentioned, the continued hammering and pounding, often from two to ten strokes on the same spot, tending to powder up the tooth under the gold. These little disintegrated particles being washed out the gold appears to draw away from the walls of the cavity, and recurrent decay sets in. Another cause is overhanging fillings under the gum margin detected only by passing a thin, scythe-shaped instrument under the gum when it will catch on the shoulder of the overhanging filling with the inevitable result—*failure.*

Dr. E. C. Chisholm (Tuscaloosa, Ala.) next read a paper entitled, "Philosophical Ideas in Operative Dentistry," illustrated by diagrams. In this short paper he made application of two well-known principles in natural science in explanation of certain well-known difficulties encountered in anchoring fillings in teeth which are elongated and somewhat loosened in their sockets, or with no other teeth touching them, as is often found in advancing age or in the incipient ravages of pyorrhœa alveolaris. The paper was illustrated by a series of diagrams. In the operation of filling such a tooth as has been described, the instrument, the particles of gold in the filling and the opposite walls of the cavity are compared to a series of suspended balls numbered from 1 to 6, the instrument being No. 1, the particles of gold 2 to 5, the wall of the cavity No. 6. In the case of the balls it is a well known law that if No. 1 be raised and let fall upon No. 2 no apparent effect will be seen in Nos. 2, 3, 4 and 5, which will remain absolutely immovable while No. 6 will fly off from No. 5 as if it alone had been struck. In the operation of filling the elongated loosened tooth the blows of the mallet upon the instrument, or No. 1 of the series, will pass through the particles of gold forming the unmoved series 2 to 5, and be perceptible only in the terminal body of the series, the tooth itself, or No. 6, being forcibly driven away from the filling by the blow which was designed to impact the filling in the tooth. And the nearer the cavity, or the point upon which the blow is impacted, is to the cutting edge of the tooth, the greater will be the jar and the movement of the tooth, the power of resistance being least in the point most remote from the apex of the root—the fixed pivotal end. If the instrument does not absolutely touch the filling at the instant of the blow the result is even worse as the series is lessened while each integral in the series loses somewhat of the power imparted by the mallet. If the instrument rebounds a double stroke is given which still further aggravates the evil. If a side stroke is given upon such a tooth it will be driven away laterally and the filling already partially condensed will be loosened and moved from its bed with a tendency to ride out upon its rounded base.

The remedy for these evils is, 1st in the use of hand pressure, or 2nd in properly supporting the tooth either by interposing a solid body between the loosened tooth and its neighbor, or by the use of Dr. E. Parmly Brown's clamps which brace several teeth together with inside and outside supporting bars. If the tooth stands alone it may be supported by the handle of a heavy instrument, which becomes the terminal body in the series, the filling and the tooth constituting the immovable numbers of the series.

Dr. Chisholm emphasized the statement that he regarded the mallet as *the only proper mode for the condensation* of gold fillings, illustrating the results of hand pressure and the mallet by the two modes of driving a nail into a plank, by driving with a hammer or by attempting to press it in with the *handle* of the hammer. The results from the use of a crooked instrument will be even worse—unstable and unsatisfactory.

Another law of which Dr. Chisholm made application in the use of crooked and angular instruments is that *the angles of incidence and reflection are the same*—the application of the principle being obvious though not easy to describe without the aid of a diagram.

Dr. Francis Peabody (Louisville, Ky.) read a paper entitled, "The Application of Vapor under Pressure for Diseased Tissue." This is a new method of treating pulpless and diseased teeth. An appliance consisting of a small metallic cylinder, through which passes a metal tube, one extremity having an ordinary syringe point, the other connected by a rubber tube to a syringe bulb. The cylinder is to be partly filled with coarse crystals of non-agglutinated iodoform and heated over an alcohol lamp or gas jet till the iodoform is fused. The syringe-point being placed in the root canal or pulp chamber of the dead tooth the rubber bulb is compressed and the iodoform vapor thus forced into the canal, permeating every portion of it and even filling the tubuli with a precipitate of iodoform, making a solid, insoluble filling, much more thorough than can be done with any plastic material. The whole tract of the canal and its contents are thoroughly dessicated and septic matters absolutely destroyed, the vapor passing through the tubuli and the apical foramen any inflammation or irritation existing in the peridental membrane is subdued, the tooth, if loose, being restored to a firm position in the alveolus. This method of treatment is found of special value in the treatment of blind abscess, diseases of the antrum and pyorrhœa alveolaris. The precipitate also forms a successful capping for exposed pulps. The objectional odor of iodoform may be to some extent disguised with caffine, menthol or oil of lemon. After further experiments some other drug free from this objectional feature, but equally valuable in results, will probably be discovered. Iodoform, however, used in this manner has proved to the writer to be a valuable acquisition in a number of apparently hopeless cases.

Dr. Noel next read a paper sent by Dr. Theo. F. Chupein. The paper, which was without title, embodied a number of practical points and valuable hints. In the application of the rubber dam in a case where decay has progressed far under the gum on one side of the tooth while the gum is in normal position and firmly adherent to the neck of the tooth elsewhere—to overcome this difficulty Dr. Chupein says—pack gutta-percha at the distal and mesial surfaces of the tooth—when the gum is forced away from these surfaces remove the gutta-percha and wrap gilling twine or ligature silk two or three times around the tooth pushing it well up on the neck of the tooth—replace the gutta-percha on the proximate surfaces. After a day or two the gum will be found well forced away and the dam may be applied without difficulty. In cavities that cannot be kept dry guttapercha covered with a very light film of oil of Cajeput can be packed into the wet cavity as a temporary filling, or even as a permanent filling when not subject to the attrition of mastication. When it becomes necessary to repair a large gold filling the surface of the gold may be dried and annealed as follows: A small drop tube with rubber bulb is filled with cotton lamp wick with the merest end of wick projecting from the point. Placing the point in alcohol, by pressure on the bulb sufficient alcohol is drawn in to moisten the wick. This will produce a minute, almost imperceptible flame, by means of which with very little, if any, discomfort to the patient the filling may be made so dry that fresh gold will adhere to the old filling if the surface is slightly roughened. This is the device of Dr. Beacock, of Brockville, Canada. The use of nitrate of silver in arresting decay, by applying it in powder, rubbed into the disorganized dentine, filling over it with gutta-percha, was recommended as having been successfully used.

A paper from Dr. T. H. Parramore (Hampton, Va.) on the use of Sterilized Sponge in Pulp Capping was next read by Dr. Noel.

At the meeting of the association at Old Point Comfort, in August, 1887, Dr. Parramore read a paper describing his method of capping exposed pulps by the use of a bit of sterilized sponge affording a nidus to catch and hold the bone-forming product of the pulps in position where it will protect, instead of forcing the irritating nodules of osteodentine, apparently the result of unsuccessful attempts of the pulp to protect itself. What was then scarcely but little more than a theory is now a practical success, one hundred and fifty cases closely watched during the intervening years showing but twenty-three failures. In one case, a pulp exposure upon the anterior surface, was capped by this method in 1886. In 1891 the living, healthy nerve became exposed by decay in a crown cavity, the pulp being found perfectly protected on the anterior surface. (This extracted tooth was sent for examination.) In treating by this method, the cavity is carefully prepared, all foreign matter being removed. The fingers, the dam, the cavity, the instruments are carefully sterilized with bichloride of mercury prepared as follows: Acid hydrochlorici, 1 drachm; hydr. bichloridi, 1 drachm; alcohol 1 drachm. M. Sig. Ten drops to one ounce 1–500 per cent. solution.

The sterilized sponge is not allowed to come in contact with the fingers but is held in oil silk and a small bit torn off with sterilized pliers and carefully applied at the point of exposure, and the cavity filled with oxyphosphate. When satisfied as to the result the permanent filling may be put in. Dr. Parramore says that at first he waited several weeks or even months, but he now usually fills permanently immediately, or at most, in a few days.

Dr. Wm. H. Cooke (Duston, Texas) sent a short paper giving his method of making combination fillings of oxyphosphate and amalgam in all molar and bicuspid cavities. Much frail enamel that would have to be cut away if all amalgam were used, can be saved by this method; perfect contact with the walls of the cavity is secured and the natural color of the tooth is retained, the teeth also lasting longer than when amalgam alone is used. The oxyphosphate is mixed to nearly the consistence of putty and about one-third of the cavity filled. While it is still plastic, amalgam in small pieces is introduced and burnished in with the engine burnishers, making a perfect amalgam margin.

At the last morning session a paper by Dr. J. L. Mewborn entitled, "Is it True that the Machine Mallet Pulverizes the Hidden Margins of Enamel," was, at his own request, read *by title.*

Dr. Noel exhibited and explained the merits of a variety of forms of apparatus for the administration of anæsthetics, including the Hayes apparatus, S. S. White's new inhaler for nitrous oxide gas, by the aid of which a little atmospheric air is admitted to the first few inhalations; an apparatus for the exhibition of ether used by John Wyeth; the Barr inhaler, and others.

On motion, Dr. Freeman, at the suggestion of Dr. B. Holly Smith, in courtesy to those who were obliged to leave in order to reach Niagara Falls in time for the meeting of the American Dental Association, the order of business was suspended, and the next place of meeting balloted for. The vote was unanimous for Chicago. The exact date was left to the Executive Committee, but will be fixed with reference to the World's Columbian Dental Congress.

Dr. E. S. Chisholm offered a resolution, in view of the present unsatisfactory method of conducting clinics, that "A Committee be appointed by the president, who shall arrange and revise methods, requiring all members to be comfortably seated in front of clinics, and good order be preserved during clinics, and that such clinicians be selected as are competent, both to instruct and render due explanations of each operation in its minuteness and detail." The resolution was unanimously adopted.

Dr. Chisholm said that as clinics are now conducted only a few men are benefitted; nine-tenths of the members are talking politics and telling anecdotes and losing the greater part of two days—a loss of both time and money.

Dr. Dotterer said that very much depended upon the chairman of clinics; also that each operator should bring his own instruments and everything that he expected to need.

He offered a resolution that the Clinic Committee be appointed in the same manner as the Executive Committee, to serve for one, two and three years. In this manner the man who has served on the committee for two years will be competent to act as chairman in his third year. The resolution of Dr. Dotterer was adopted and Dr. E. S. Chisholm appointed for one year, Dr. L. P. Dotterer two years and Dr. Noel three years.

Dr. Crawford said he thought he was one of the first to introduce what are known as oral clinics. Let the clinician give first a diagnosis then a prescription. This much will be sufficient for many who are proficient in manipulative details. They may retire while the actual operation is performed for the benefit of those who do not fully seize the idea from the oral demonstration.

Dr. A. P. Johnson said it was not necessary for an expert operator to watch another pick up every particle of gold and pack it away in a clinic. One oral clinic is worth more than fifty times the actual performance of the work.

Dr. Richards offered a resolution for the permanent employment of a stenographer for the association whose duty it should be to furnish a synopsis of each day's proceedings for revision by a committee before publication in the daily papers. He should be one who would make himself familiar with the technicalities of the profession.

Dr. Catching moved to amend by substituting reporter for stenographer, as some of the best reporters ever employed by the association were not stenographers.

Dr. Beach moved to amend by substituting stenographic reporter. Carried.

Dr. John C. Storey (Dallas, Texas) spoke of the work being done by the Executive Committee of the World's Columbian Dental Congress and their lack of funds, and offered a resolution giving all the unappropriated funds of the association to the treasurer of the Congress. After some discussion *pro* and *con*, the resolution was amended by substituting the definite sum of $200.00 and carried. The regular order of business was now resumed.

On motion, the subject of Operative Dentistry was passed.

The chairman of the Committee on Dental Hygiene being absent no report was presented.

The report of the Committee on Dental Histology was received, and the paper of Dr. E. P. Beadles read by title.

No report from the Committee on Dental Chemistry.

Voluntary essays received from Drs. Crews, Thackston and Chisholm, were read by title owing to the necessity for adjournment in order that members might reach Niagara in time for the American Dental Association. All papers read by title were ordered published in the transactions of the meeting, where they may be found under their proper titles, where also the reports of the Committees on Clinics and New Appliances will be found.

A letter from the venerable Dr. W. H. H. Thackston was read, and a message of greeting and thanks for his paper voted him.

The Committee on Necrology reported memorial resolutions in memory of Dr. John Allen, of New York, Dr. C. A. Kingsbury, of Philadelphia and Dr. Wm. Deason, of Mobile, Ala. A page in the minutes was ordered inscribed to their memory.

Dr. E. C. Kirk, editor of the *Dental Cosmos*, then addressed the Association briefly, on the value of systematic work in the associations, and outlined a plan now being agitated in the American Association. A certain number of pertinent questions upon weighty topics, to be formulated and distributed among State and local societies, to form the basis of discussion during the year. A report of these discussions, being kept by the secretaries of the different societies, is to be forwarded to the chairmen of sections of the larger bodies. A digest of these reports will be reported to the body as a whole, and will offer material for discussion in that body, embodying the views of the profession all over the country. This must create an interest in the governing body, while each local society will feel that it is in touch with the parent organization, and delegates will be sent to see that local interests are looked after. This is not designed to interfere with the regular set papers, but as an additional feature, in order to get the co-operation of the whole dental body in association work. From this we would be able to formulate a system of practice wholly American. He concluded by saying:

"Dental literature is in your hands; you make it; the journals only disseminate. I hope the plan thus briefly outlined will meet with your favor, either in whole or in part."

After the usual votes of thanks to the president, the railroads, the hotel and the ladies, and the formal installation in office of the officers-elect,—the president by proxy, he having left earlier in the day,—the Association adjourned to meet in Chicago in 1893—exact date left to the executive committee for future announcement.

